# *Nepenthes* Extract Induces Selective Killing, Necrosis, and Apoptosis in Oral Cancer Cells

**DOI:** 10.3390/jpm11090871

**Published:** 2021-08-31

**Authors:** Kun-Han Yang, Jen-Yang Tang, Yan-Ning Chen, Ya-Ting Chuang, I-Hsuan Tsai, Chien-Chih Chiu, Li-Jie Li, Tsu-Ming Chien, Yuan-Bin Cheng, Fang-Rong Chang, Ching-Yu Yen, Hsueh-Wei Chang

**Affiliations:** 1 Graduate Institute of Natural Products, Kaohsiung Medical University, Kaohsiung 80708, Taiwan; R100024@kmu.edu.tw; 2 School of Post-Baccalaureate Medicine, Kaohsiung Medical University, Kaohsiung 80708, Taiwan; reyata@kmu.edu.tw; 3 Department of Radiation Oncology, Kaohsiung Medical University Hospital, Kaohsiung 80708, Taiwan; 4 Department of Biomedical Science and Environmental Biology, College of Life Science, Kaohsiung Medical University, Kaohsiung 80708, Taiwan; u107023010@gap.kmu.edu.tw (Y.-N.C.); u107023007@gap.kmu.edu.tw (Y.-T.C.); s0932961465@gmail.com (I.-H.T.); u103023023@kmu.edu.tw (L.-J.L.); 5 Department of Biotechnology, Kaohsiung Medical University, Kaohsiung 80708, Taiwan; cchiu@kmu.edu.tw; 6 Graduate Institute of Clinical Medicine, College of Medicine, Kaohsiung Medical University, Kaohsiung 80708, Taiwan; u108801005@kmu.edu.tw; 7 Department of Urology, School of Medicine, College of Medicine, Kaohsiung Medical University, Kaohsiung 80708, Taiwan; 8 Department of Urology, Kaohsiung Medical University Hospital, Kaohsiung 80708, Taiwan; aaronfrc@kmu.edu.tw; 9 Department of Marine Biotechnology and Resources, National Sun Yat-sen University, Kaohsiung 80424, Taiwan; jmb@mail.nsysu.edu.tw; 10 Department of Oral and Maxillofacial Surgery Chi-Mei Medical Center, Tainan 71004, Taiwan; 11 School of Dentistry, Taipei Medical University, Taipei 11031, Taiwan; 12 Center for Cancer Research, Kaohsiung Medical University, Kaohsiung 80708, Taiwan

**Keywords:** *Nepenthes*, preferential killing, necrosis, apoptosis, oxidative stress, oral cancer

## Abstract

Ethyl acetate *Nepenthes* extract (EANT) from *Nepenthes thorellii × (ventricosa × maxima)* shows antiproliferation and apoptosis but not necrosis in breast cancer cells, but this has not been investigated in oral cancer cells. In the present study, EANT shows no cytotoxicity to normal oral cells but exhibits selective killing to six oral cancer cell lines. They were suppressed by pretreatment of the antioxidant inhibitor *N*-acetylcysteine (NAC), demonstrating that EANT-induced cell death was mediated by oxidative stress. Concerning high sensitivity to EANT, Ca9-22 and CAL 27 oral cancer cells were chosen for exploring detailed selective killing mechanisms. EANT triggers a mixture of necrosis and apoptosis as determined by annexin V/7-aminoactinmycin D analysis. Still, they show differential switches from necrosis at a low (10 μg/mL) concentration to apoptosis at high (25 μg/mL) concentration of EANT in oral cancer cells. NAC induces necrosis but suppresses annexin V-detected apoptosis in oral cancer cells. Necrostatin 1 (NEC1), a necroptosis inhibitor, moderately suppresses necrosis but induces apoptosis at 10 μg/mL EANT. In contrast, Z-VAD-FMK, a pancaspase inhibitor, slightly causes necrosis but suppresses apoptosis at 10 μg/mL EANT. Furthermore, the flow cytometry-detected pancaspase activity is dose-responsively increased but is suppressed by NAC and ZVAD, although not for NEC1 in oral cancer cells. EANT causes several oxidative stress events such as reactive oxygen species, mitochondrial superoxide, and mitochondrial membrane depolarization. In response to oxidative stresses, the mRNA for antioxidant signaling, such as nuclear factor erythroid 2-like 2 (*NFE2L2*), catalase (*CAT*), heme oxygenase 1 (*HMOX1*), and thioredoxin (*TXN*), are overexpressed in oral cancer cells. Moreover, EANT also triggers DNA damage, as detected by γH2AX and 8-oxo-2′-deoxyguanosine adducts. The dependence of oxidative stress is validated by the evidence that NAC pretreatment reverts the changes of cellular and mitochondrial stress and DNA damage. Therefore, EANT exhibits antiproliferation involving an oxidative stress-dependent necrosis/apoptosis switch and DNA damage in oral cancer cells.

## 1. Introduction

Oral cancer shows high morbidity and mortality worldwide [1]. In addition to surgery, chemo-radiotherapy is an alternative treatment, but it is widely associated with side effects [2]. It may partly attribute to the adverse impact of harming normal cells, which limits its application for cancer therapy. Therefore, it is crucial to identify preferentially killing drugs against cancer cells with a low side effect on normal cells.

Multi-target cancer therapies have become a common strategy. Natural products containing several bioactive components exhibit a multiple targeting potential [3,4,5]. For example, *Nepenthes* plants, the traditional herbal medicine in Southeast Asia [6], contain several bioactive compounds, including flavonoids [7], naphthalene glucosides [8], naphthoquinones [9], steroids [10], triterpenoids [10], and polyphenol [11].

Anticancer effects of extracts of the pitcher plant *Nepenthes* were recently reported. For example, the methanol extract of *N. alata* Blanco exhibits an anti-breast cancer effect [12]. The ethyl acetate extract of *N. thorellii ×* (*ventricosa × maxima*) (EANT) also shows antiproliferation to breast cancer cells [13]. However, the antiproliferation of oral cancer cells remains unclear.

Several modes of cell death may attribute to apoptosis, necrosis, and necroptosis. Apoptotic cells are characterized by cell shrinkage, membrane blebbing, formation of apoptotic bodies, chromatin condensation, and DNA fragmentation [14]. Necrotic cells are characterized by cell swelling and poor plasma membrane integrity [14]. Necroptosis is a programmed type of necrosis [15]. Although they exhibit distinct mechanisms, apoptosis and necrosis are partly interrelated [16]. Moreover, drug-induced cell death is sometimes confusing, and the cell death types may vary with cell line or dose.

In the present study, the preferential killing effects and mechanisms of ethyl acetate extract EANT were investigated. Moreover, the interchange of cell death mechanisms (necrosis and apoptosis), oxidative stress, and DNA damage responses by different doses of EANT were also examined.

## 2. Materials and Methods

### 2.1. EANT Preparation and Reagents

The EANT preparation and its HPLC fingerprint profile were described in our previous study [13]. Briefly, EANT was prepared from the methanol soaking and water/ethyl acetate partition of the aerial parts of *N. thorellii* *× (ventricosa* *× maxima)*, containing several bioactive components such as plumbagin, *cis*-isoshinanolone, quercetin 3-*O*-(6″-*n*-butyl β-D-glucuronide), and fatty acids [13]. EANT was immediately dissolved in dimethyl sulfoxide (DMSO) before treatment.

The impacts of oxidative stress, apoptosis, and necroptosis were assessed by pretreating for 1 h with 2 mM *N*-acetylcysteine (NAC) (Sigma-Aldrich; St. Louis, MO, USA) [17,18,19,20], 1 h with 50 μM necrostatin-1 (NEC1) (TargetMol; Wellesley, MA, USA) [21], and 2 h with 100 μM Z-VAD-FMK (ZVAD) [22] (Selleckchem.com; Houston, TX, USA).

### 2.2. Cell Lines and Cell Viability Assay

ATCC cell lines (Manassas, VA, USA) including oral cancer (CAL 27, OC-2, and SCC9) and normal (HGF-1) cell lines, as well as RIKEN BioResource Research Center cell lines (Tsukuba, Ibaraki, Japan) including oral cancer cells (HSC-3 and Ca9-22) were used. In addition, another oral cancer cell line (OECM-1) [23] was kindly provided by Dr. Wan-Chi Tsai in Kaohsiung Medical University, Taiwan. All detailed culture conditions were previously described [24]. Cell viability was assessed by the mitochondrial metabolism-based MTS kit (Promega Corporation, Madison, WI, USA) [25].

### 2.3. Cell Cycle Assay

Following fixation, cells were stained with 7-aminoactinmycin D (7AAD) (1 μg/mL, 30 min) (Biotium; Hayward, CA, USA) [26] for measuring cellular DNA content. This content was applied to cell cycle phase determination by performing Accuri C6 flow cytometry (Becton-Dickinson, Mansfield, MA, USA).

### 2.4. Apoptotic and Necrotic Flow Cytometry Assays

Apoptosis and necrosis were assessed by annexin V/7AAD [27,28,29] (Strong Biotech; Taipei, Taiwan), and apoptosis signaling was detected by the generic pancaspase (caspases-1 and 3 to 9) activity methods [25] (Abcam, Cambridge, UK) according to their commercial protocols. In addition, their intensities were monitored by Accuri C6 flow cytometry. For the annexin V/7AAD method, the criterion to determine the necrosis and apoptosis was described as follows: necrosis: 7AAD (+)/annexin V (−); early/late apoptosis: 7AAD (−)/annexin V (+) and 7AAD (+)/annexin V (+). Moreover, apoptosis was also detected by the Caspase-Glo^®^ 3/7 system (Promega; Madison, WI, USA) as described [30].

### 2.5. Oxidative Stress-Detected Flow Cytometry Assays

Oxidative stresses were assessed by increasing reactive oxygen species (ROS)/mitochondrial superoxide (MitoSOX) and decreasing mitochondrial membrane potential (MMP). 2′,7′-dichlorodihydrofluorescein diacetate (DCFH-DA; Sigma-Aldrich) [31] is the ROS detecting probe, and the optimal condition is 10 μM for 30 min. MitoSOX™ Red [32] (50 nM, 30 min) is the mitochondrial superoxide-specific detecting probe. DiOC_2_(3) (5 nM, 30 min) [33] (Invitrogen; San Diego, CA, USA) is a MMP detecting probe. In addition, their intensities were monitored by Accuri C6 flow cytometry.

### 2.6. Antioxidant Gene Expression Assay

Trizol solution (Invitrogen) and OmniScript RT kit (Qiagen, Valencia, CA, USA) were respectively applied to extract RNA and convert it to cDNA [34]. A real-time PCR performing touch-down program [35] was used to quantitate the mRNA expressions for a panel of antioxidant genes [36,37] in reference to the internal control, which was the *GAPDH* gene. The primer information for nuclear factor erythroid 2-like 2 (*NFE2L2*), catalase (*CAT*), heme oxygenase 1 (*HMOX1*), and thioredoxin (*TXN*) was described in detail [38].

### 2.7. DNA Damage-Detected Flow Cytometry Assays

Both γH2AX and 8-hydroxy-2-deoxyguanosine (8-OHdG) were chosen as cellular DNA damage markers. Following fixation, the γH2AX [39] and 8-OHdG [40] expressions were detected by antibody-based methods. For γH2AX measurement, Santa Cruz Biotechnology γH2AX antibody (Santa Cruz, CA, USA) (4 °C, 1 h) and Alexa Fluor^®^488-linked secondary antibody (Cell Signaling Technology) were sequentially applied to fixed cells, and counterstained with 7AAD (5 μg/mL, 30 min). For 8-OHdG measurement, the 8-OHdG-FITC antibody (Santa Cruz Biotechnology) (4 °C, 1 h) was applied. In addition, their intensities were monitored by Accuri C6 flow cytometry.

### 2.8. Statistical Analysis

Except for the NAC effect on EANT-treated cells, for which a 24 h MTS assay was compared applying a Student’s t-test), the remaining experiments were analyzed by one-way analysis of variance (ANOVA) with a *post hoc* test for multiple comparisons. Data top-labeled with different letters show significant differences (*p* < 0.05). The examples for illustrating the statistical analysis are provided in some figure legends.

## 3. Results

### 3.1. EANT Induces Selective Killing of Oral Cancer Cells via Oxidative Stress

**Figure 1:**
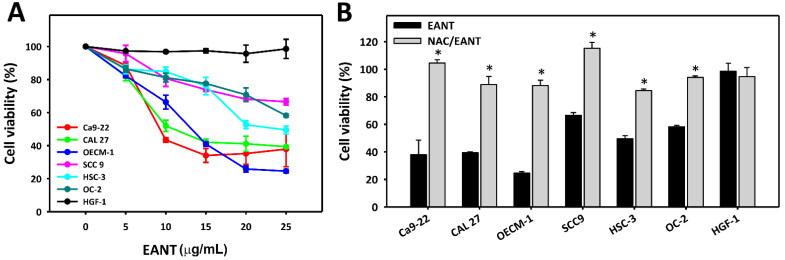
Cell viability. (**A**) Cell viability for several oral cancer cell lines and normal oral (HGF-1) cell lines in EANT treatments for 24 h MTS assay. (**B**) NAC (2 mM) pretreatment outcome of EANT treatments (0 and 25 μg/mL) for 24 h MTS assay. The cell viability of untreated controls for each oral cancer cell line was adjusted to 100%, which is not shown here. Data (means ± SD, *n* = 3) showing * differ in significance for paired comparisons (*p* < 0.05).

EANT shows high cytotoxicity to several oral cancer cell lines (Ca9-22, CAL 27, OECM-1, SCC9, HSC-3, and OC-2), but it shows no cytotoxicity to a normal oral cell line (HGF-1) (Figure 1A). NAC pretreatment (Figure 1B) recovered these cytotoxicities in oral cancer cells, demonstrating that EANT induces selective killing in oral cancer cells via oxidative stress. Here, Ca9-22 and CAL 27 cells showed here high sensitivity, and they were chosen for the following experiments.

### 3.2. EANT Induces SubG1 Accumulation in Oral Cancer Cells Depending on Oxidative Stress

**Figure 2:**
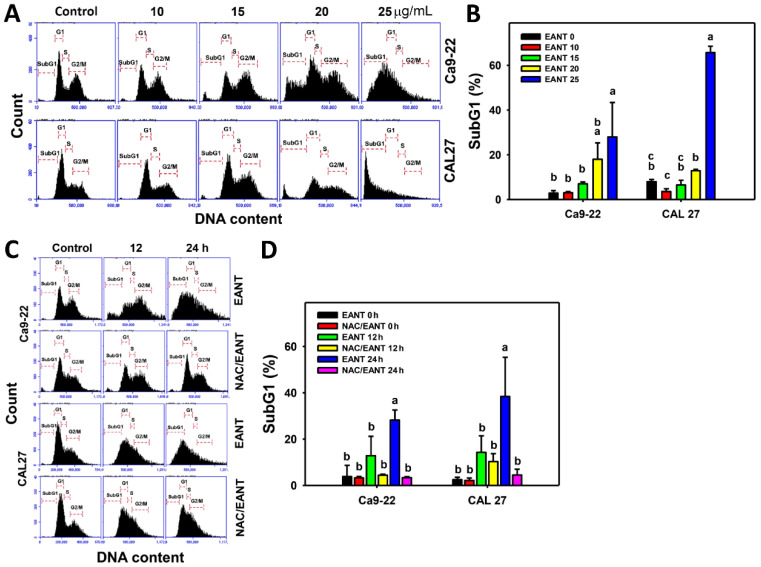
Cell cycle analysis. (**A**,**B**) Cell cycle patterns and statistical analysis for EANT treated oral cancer cells (CAL 27 and Ca9-22) over 24 h. (**C**,**D**) NAC (2 mM) pretreatment outcome to cell cycle patterns and statistical analysis in EANT treatments (0 and 25 μg/mL) for 12 and 24 h. Data (means ± SD, *n* = 3) with different letters on-top indicate significant differences for multiple comparisons (*p* < 0.05). One-way analysis of variance (ANOVA) with a *post hoc* test was applied to multiple comparisons. For example (Ca9-22 cells in Figure 2B), the EANT 0, EANT 10, EANT 15, and EANT 20 show “b and ba”, indicating nonsignificant differences between each other because they overlap with the same lowercase letter “b”. Similarly, the EANT 20 and EANT 25 show “ba” and “a”, indicating nonsignificant differences between each other because they overlap with the same lowercase letter “a”. In contrast, EANT 0, EANT 10, and EANT 15 show “b” and EANT 25 shows “a” indicating significant differences among each other because they do not overlap with the same lowercase letter.

EANT increases the subG1 population of two oral cancer cell lines (Figure 2A,B). Furthermore, the subG1 accumulations in oral cancer cells in a time-dependent manner were reduced by NAC (Figure 2C,D), revealing that EANT induces subG1 accumulation in oral cancer cells mediated by oxidative stress.

### 3.3. EANT Differentially Induces Necrosis and Apoptosis in Oral Cancer Cells Depending on Oxidative Stress

**Figure 3:**
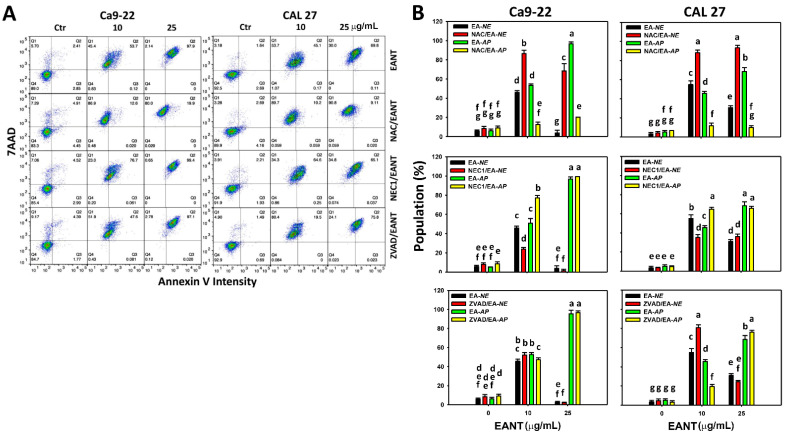
Annexin V/7AAD analysis. Q1, Q2, Q3, and Q4 marked in each panel indicate the necrosis, late apoptosis, early apoptosis, and lived cells, respectively. EA, EANT; *NE*, necrosis; *AP*, apoptosis (Q2 + Q3). For example, EA-*NE* and NAC/EA-*NE* indicate the necrosis (*NE*) status for EANT (EA) treatment in the absence and presence of NAC, respectively. EA-*NE* and NEC1/EA-*NE* indicate the *NE* status for EA treatment in the absence and presence of NEC1, respectively. EA-*NE* and ZVAD/EA-*NE* indicate the *NE* status for EA treatment in the absence and presence of ZVAD, respectively. (**A**,**B**) Annexin V/7AAD pattern and statistical analysis. With the inhibitor pretreatments for oxidative stress, necrosis, and apoptosis (10 mM NAC for 1 h, 50 μM NEC1 for 1 h, and 100 μM ZVAD for 2 h), or not, oral cancer cells (Ca9-22 and CAL 27) were treated with EANT (control, 10, 25 μg/mL) for 24 h. Data (mean ± SD, *n* = 3) showing different letters on top differ significantly for multiple comparisons (*p* < 0.05). One-way analysis of variance (ANOVA) with a *post hoc* test was applied to multiple comparisons. For example (Ca9-22 cells in Figure 3B), the EN-*NE* (red color) at 0, 10, and 25 μg/mL show “fg, b, and c”, indicating significant differences among each other because they do not overlap with the same lowercase letter. Similar to 25 μg/mL EANT, EA-*NE*, NAC/EA-*NE*, EA-*AP*, and NAC/EA-*AP* show “g, c, a, and e”, indicating significant differences. In contrast, EA-*NE*, NAC/EA-*NE*, EA-*AP*, and NAC/EA-*AP* at the control (0 EANT) show “fg”, indicating nonsignificant differences between each other because they overlap with the same lowercase letter “fg”.

The apoptosis and necrosis of oral cancer cells following EANT treatment were assessed using the flow cytometry-based annexin V/7AAD method [28,41,42] (Figure 3A). For Ca9-22 and CAL 27 cells, EANT at a low concentration (10 μg/mL) induces a mixture of necrosis (Q1 in annexin V/7AAD) and apoptosis (Q2/Q3 in annexin V/7AAD) (Figure 3B). EANT at a high concentration (25 μg/mL) mainly induces apoptosis (Q2/Q3). Accordingly, EANT at higher concentration partly induces the switch of necrosis to apoptosis.

NAC [17,18,19,20] and ZVAD [22] are inhibitors for oxidative stress and apoptosis. NEC1, the receptor-interacting with the serine/threonine-protein kinase 1 (RIPK1) inhibitor, can suppress necroptosis [21]. To elucidate the contribution of oxidative stress, necroptosis, and apoptosis, these inhibitors were applied in EANT treated oral cancer cells.

At low and high concentrations of EANT, the NAC effect to EANT-treated oral cancer cells (Ca9-22 and CAL 27) shows that NAC pretreatment and EANT post-treatment (NAC/EANT) can induce necrosis (NAC/EA-*NE* (EANT-necrosis) vs. EA-*NE*) and suppress apoptosis (NAC/EA-*AP* (EANT-apoptosis) vs. EA-*AP*) (Figure 3, top). Accordingly, EANT at low and high concentrations differentially triggers necrosis and apoptosis in oral cancer cells mediated by oxidative stress.

For the NEC1 effect in Ca9-22 and CAL 27 cells at low concentrations of EANT (Figure 3, middle), NEC1/EANT moderately suppresses necrosis (NEC1/EA-*NE* vs. EA-*NE*) and induces apoptosis (NEC1/EA-*AP* vs. EA-*AP*). In Ca9-22 and CAL 27 cells at high concentrations of EANT, NEC1/EANT shows no or only a slight change to necrosis and apoptosis. Accordingly, NEC1 switches EANT-induced necrosis to apoptosis in oral cancer cells at low concentrations of EANT.

For the ZVAD effect in Ca9-22 cells at low concentrations of EANT (Figure 3, bottom), ZVAD/EANT slightly induces necrosis (ZVAD/EA-*NE* vs. EA-*NE*) and suppresses apoptosis (ZVAD/EA-*AP* vs. EA-*AP*). In CAL 27 cells at low concentrations of EANT, ZVAD/EANT moderately induces necrosis (ZVAD/EA-*NE* vs. EA-*NE*) and suppresses apoptosis (ZVAD/EA-*AP* vs. EA-*AP*). In Ca9-22 and CAL 27 cells at high concentrations of EANT, ZVAD/EANT shows no or only a slight change to necrosis and apoptosis. Accordingly, ZVAD switches EANT-induced apoptosis to necrosis in oral cancer cells at low concentrations of EANT.

### 3.4. EANT Induces Pancaspase Activation for Apoptosis to Oral Cancer Cells Depending on Oxidative Stress

**Figure 4:**
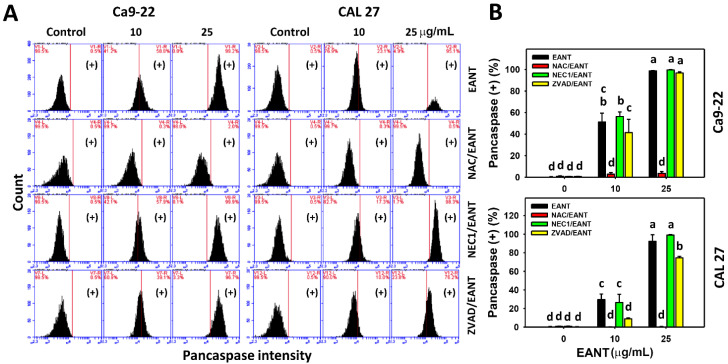
Pancaspase activation analysis. (**A**,**B**) Pancaspase flow cytometry and statistics. With the inhibitor pretreatments for oxidative stress, necrosis, and apoptosis (10 mM NAC for 1 h, 50 μM NEC1 for 1 h, and 100 μM ZVAD for 2 h), or not, oral cancer cells (Ca9-22 and CAL 27) were treated with EANT (control, 10, 25 μg/mL) for 24 h. Data (mean ± SD, *n* = 3) showing different letters on-top differ significantly for multiple comparisons (*p* < 0.05).

The pancaspase activation for apoptosis in oral cancer cells following EANT treatments was assessed by the flow cytometry-based pancaspase method (Figure 4A). For Ca9-22 and CAL 27 cells, EANT moderately induces apoptosis at a low concentration (10 μg/mL) and dramatically induces apoptosis EANT at a high concentration (25 μg/mL).

At low and high concentrations of EANT, the NAC effect to EANT-treated oral cancer cells (Ca9-22 and CAL 27) shows that the combination of NAC pretreatment and EANT post-treatment (NAC/EANT) suppresses apoptosis compared to EANT alone (Figure 4B). Accordingly, EANT triggers pancaspase activation in oral cancer cells mediated by oxidative stress.

For the NEC1 effect (Figure 4B), NEC1/EANT does not change pancaspase activity compared to EANT alone at low and high concentrations of EANT in both Ca9-22 and CAL 27 cells. For the ZVAD effect (Figure 4B), ZVAD/EANT moderately suppresses pancaspase activity compared to EANT alone at low concentrations of EANT in both Ca9-22 and CAL 27 cells. For a high concentration of EANT, ZVAD/EANT does not change the pancaspase activity of Ca9-22 cells, while ZVAD/EANT moderately suppresses the pancaspase activity of CAL 27 cells.

### 3.5. EANT Induces Selecitve ROS Generation in Oral Cancer Cells via Oxidative Stress

**Figure 5:**
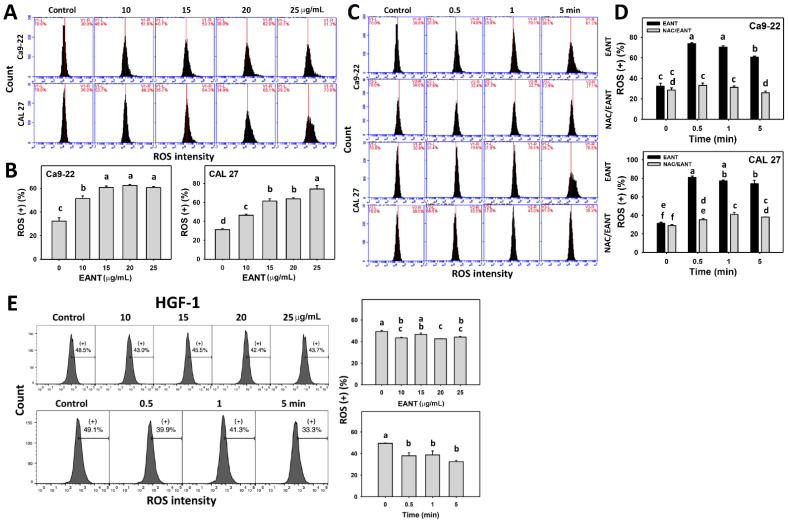
ROS analysis. (**A**,**B**) ROS pattern and statistical analysis for EANT treated oral cancer cells (CAL 27 and Ca9-22) for 5 min. (**C**,**D**) NAC (10 mM) pretreatment outcome to ROS patterns and statistical analysis in EANT treatments (0 and 25 μg/mL) for 0, 0.5, 1, and 5 min. (**E**) ROS pattern and statistical analysis for EANT treated normal oral (HGF-1) cells for different concentrations (0, 10, 15, 20, and 25 μg/mL for 5 min) and treatment times (25 μg/mL for 0, 0.5, 1, and 5 min) of EANT. Data (means ± SD, *n* = 3) showing different letters on-top differ significantly for multiple comparisons (*p* < 0.05).

EANT shows a high ROS-positive population in two oral cancer cell lines (Figure 5A,B). The ROS generation in oral cancer cells was recovered by NAC (Figure 5C,D), suggesting that EANT induces ROS in oral cancer cells via oxidative stress. In contrast, EANT does not induce ROS generation for normal oral cells (Figure 5E) at the same conditions (concentration and time) as it does in oral cancer cells.

### 3.6. EANT Induces MitoSOX Generation Depending on Oral Cancer Cells

**Figure 6:**
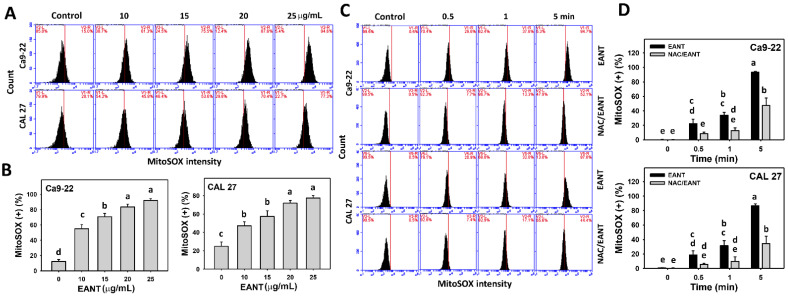
MitoSOX analysis. (**A**,**B**) MitoSOX patterns and statistical analysis for EANT treated oral cancer cells (CAL 27 and Ca9-22) for 5 min. (**C**,**D**) NAC (2 mM) pretreatment outcome to MitoSOX patterns and statistical analysis in EANT treatments (0 and 25 μg/mL) for 0, 0.5, 1, and 5 min. Data (mean ± SD, *n* = 3) showing different letters on-top differ significantly for multiple comparisons (*p* < 0.05).

EANT shows a high percentage of MitoSOX-positive population (Figure 6A,B) in two oral cancer cell lines. This MitoSOX generation (Figure 6C,D) in oral cancer cells was recovered by NAC, suggesting that EANT induces MitoSOX generation in oral cancer cells mediated by oxidative stress.

### 3.7. EANT Induces MMP Depletion Depending on Oral Cancer Cells

**Figure 7:**
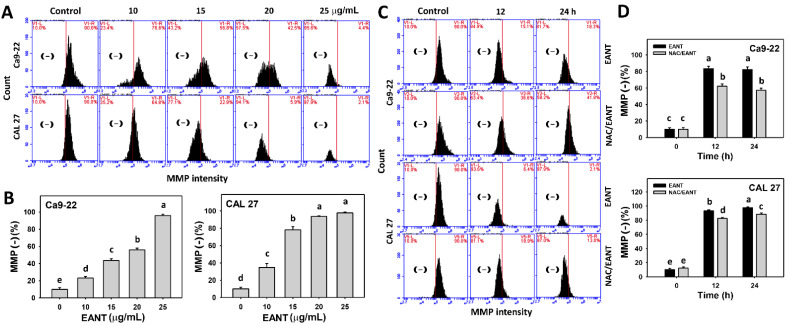
MMP analyses. (**A**,**B**) MMP patterns and statistical analysis for EANT treated oral cancer cells (CAL 27 and Ca9-22) for 24 h. (**C**,**D**) NAC (10 mM) pretreatment outcome on MMP patterns and statistical analysis in EANT treatments (0 and 25 μg/mL) for 0, 12, and 24 h. Data (mean ± SD, *n* = 3) showing different letters on top differ significantly for multiple comparisons (*p* < 0.05).

EANT shows a high proportion of MMP negative population (Figure 7A,B) in two oral cancer cell lines. This MMP destruction (Figure 7C,D) in oral cancer cells were recovered by NAC, suggesting that EANT induces MMP destruction in oral cancer cells mediated by oxidative stress.

### 3.8. EANT Upregulates mRNA Expression for Antioxidant Genes in Oral Cancer Cells

**Figure 8:**
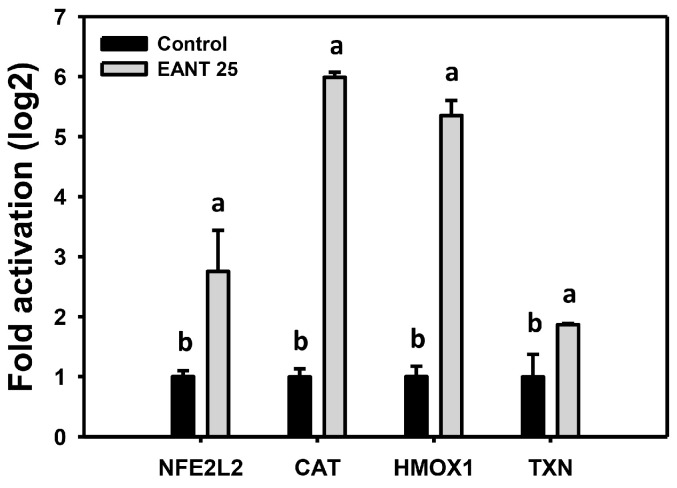
Antioxidant gene expression in EANT treated oral cancer cells. Cells were treated with EANT (control and 25 μg/mL (EANT 25) for 24 h), respectively. Several antioxidant genes were chosen for real-time RT-PCR analysis as indicated. Data (mean ± SD, *n* = 3) showing different letters on-top differ significantly for multiple comparisons (*p* < 0.05).

The antioxidant system commonly changes in response to oxidative stress [43,44]. The mRNA expressions of antioxidant genes such as *NFE2L2*, *CAT*, *HMOX1*, and *TXN,* were tested. EANT differentially induces these mRNA expressions (Figure 8).

### 3.9. EANT Induces γH2AX in Oral Cancer Cells Depending on Oxidative Stress

**Figure 9:**
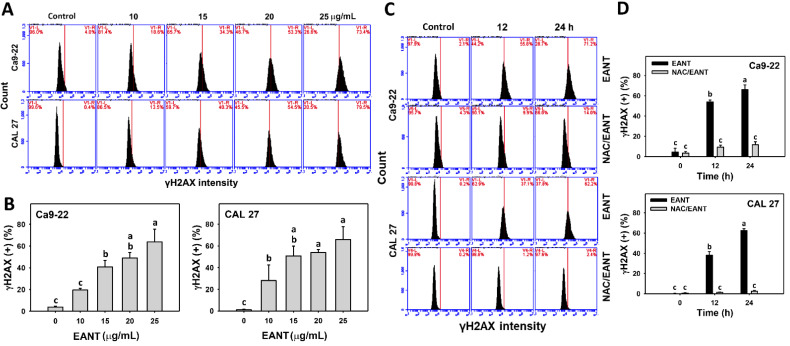
γH2AX analysis. (**A**,**B**) γH2AX pattern and statistical analysis for EANT treated oral cancer cells (CAL 27 and Ca9-22) for 24 h. (**C**,**D**) NAC (2 mM) pretreatment outcome on γH2AX patterns and statistical analysis in EANT treatments (0 and 25 μg/mL) for 12 and 24 h. Data (means ± SD, *n* = 3) showing non-overlapping alphabets differ significantly for multiple comparisons (*p* < 0.05).

EANT shows a high percentage of γH2AX positive populations in two oral cancer cell lines (Figure 9A,B). These γH2AX in oral cancer cells were recovered by NAC (Figure 9C,D), suggesting that EANT induces γH2AX DNA damage in oral cancer cells mediated by oxidative stress.

### 3.10. EANT Induces 8-OHdG in Oral Cancer Cells Depending on Oxidative Stress

**Figure 10:**
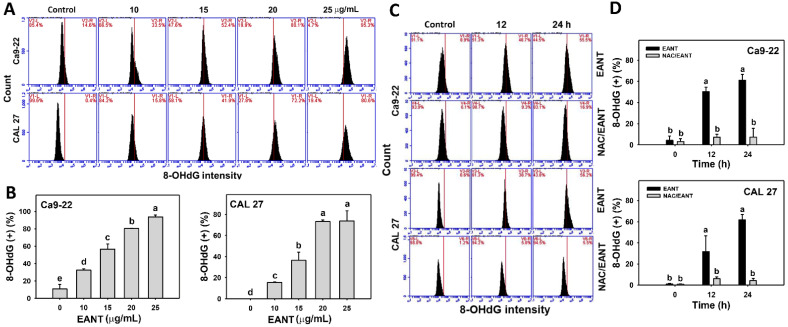
8-OHdG analysis. (**A**,**B**) 8-OHdG pattern and statistical analysis for EANT-treated oral cancer cells (CAL 27 and Ca9-22) for 24 h. (**C**,**D**) NAC (2 mM) pretreatment outcome on 8-OHdG pattern and statistical analysis in EANT treatments (0 and 25 μg/mL) for 12 and 24 h. Data (means ± SD, *n* = 3) showing different letters on top differ significantly for multiple comparisons (*p* < 0.05).

EANT shows a high percentage of the 8-OHdG-positive population in two oral cancer cell lines (Figure 10A,B). This 8-OHdG induction in oral cancer cells was recovered by NAC (Figure 10CD), suggesting that EANT induces 8-OHdG DNA damage in oral cancer cells mediated by oxidative stress.

## 4. Discussion

The antiproliferation effect of ethyl acetate extract of EANT has been reported in breast cancer cells [13] but not for oral cancer cells. The cell death modes, such as necrosis and apoptosis, maybe partly be interrelated [16]. However, necrosis does not appear in EANT-treated breast cancer cells. Therefore, necrosis and apoptosis changes in EANT-treated oral cancer cells were examined in the present study. The preferential killing effects and mechanisms for the interchange of cell death mechanisms (necrosis and apoptosis), oxidative stress, and DNA damage responses by different doses of EANT are discussed below.

### 4.1. EANT Inhibits Proliferation of Oral Cancer Cells but Not of Normal Oral Cells

Previously, we reported that EANT inhibited the proliferation of breast cancer cells, i.e., IC_50_ values for MCF7 (15 μg/mL) and SKBR3 (25 μg/mL) during an 24 h MTS assay [13]. In the current study, oral cancer Ca9-22, CAL 27, OECM-1, and HSC-3 cells show IC_50_ values of 9.27, 11.05, 13.2, and 24 μg/mL, respectively, following a 24 h EANT treatment by MTS assay. However, this result was based on the short-term exposure (24 h). It warrants detailed investigation for the MTS assays of long-term exposures to Ca9-22 and CAL 27 cells following the 72 and 96 h EANT treatment in the future.

In general, EANT demonstrates a more sensitive antiproliferation effect in oral cancer cells than breast cancer cells. Moreover, EANT shows selective killing to breast [13] and oral cancer cells (Figure 1) but offers little or no cytotoxicity to normal breast [13] and oral cells, showing that EANT has drug safety. This selective killing effect of antiproliferation in oral cancer cells may be helpful for anticancer therapy, but it still lacks the in vivo evidence. It warrants in vivo experiments to confirm the effects of EANT on oral cancer treatment in the future.

Three main compounds, such as plumbagin, *cis*-isoshinanolone, and quercetin 3-*O*-(6**″**-*n*-butyl β-D-glucuronide), were identified from EANT by HPLC fingerprinting, [13]. These compounds may contribute to the cell-killing effect of EANT on several types of cancer cells. For example, plumbagin shows IC_50_ values for 0.26 and 0.2 μg/mL to leukemia HL-60 and K-562 cells in 24 h trypan-blue assays [45], but provides IC_50_ values of 1.16 μg/mL to breast cancer MCF7 cells in a 24 h MTT assay [12]. *cis*-isoshinanolone [46] and quercetin 3-*O*-(6**″**-*n*-butyl β-D-glucuronide) [47] exhibit an IC_50_ of 15.76 and 61.4 μg/mL, respectively, to breast cancer MCF-7 cells in 72 h MTT and sulphorhodamine-B assays.

### 4.2. EANT Produces Oxidative Stress and Triggers an Antixodant Response in Oral Cancer Cells

Anticancer drugs with an oxidative stress-modulating function commonly induce preferential anticancer effects [48]. For example, sinularin generates oxidative stress and exhibits selective killing of breast cancer cells [28]. EANT produces cellular and mitochondrial stress in breast cancer cells [13], but the changes for antioxidant signaling and MMP have not been investigated. Oxidative stress and mitochondrial dysfunction (MMP destruction) occur in EANT-treated oral cancer cells (Figure 5 and Figure 6). Moreover, EANT does not increase or induce oxidative stress in noncancer cells (Figure 5E). Therefore, EANT selectively induces oxidative stress in oral cancer cells.

In response to oxidative stress, the antioxidant CAT was activated [43]. In UVC-irradiated mice, oxidative stress was generated, and both mRNA and protein expressions for *CAT* and *HMOX1* genes were upregulated [49]. Moreover, oxidative stress can activate NFE2L2 to target and activate TXN expression [50].

In the present study, EANT generates oxidative stress (Figure 5 and Figure 6), which is accompanied by mRNA overexpression for antioxidant genes (*NFE2L2*, *CAT*, *HMOX1*, and *TXN*) (Figure 7) in oral cancer cells. Accordingly, EANT induces antioxidant gene expression but is unable to reduce oxidative stress in oral cancer cells.

### 4.3. EANT Triggers Concentration-Dependant Necrosis and Apoptosis Switches in Oral Cancer Cells

The dysregulation between oxidants and antioxidants can cause necrosis and apoptosis [51,52]. Apoptosis and necrosis are interrelated, although they have different underlying mechanisms. Necrosis and apoptosis switches were reported in several studies. For example, apoptosis and necrosis in pancreatic acinar cells are interchangeable during acute pancreatitis [16]. Pancreatic acinar cells in the acute pancreatitis model trigger apoptosis, but switch to necrosis by apoptosis inhibition [53,54,55]. Prothymosin-α1 inhibits necrosis and induces apoptosis in cultured neurons. *PKCβII* knockdown suppresses this cell death (necrosis/apoptosis) switch [56]. Ionizing radiation (IR) shows a dose-dependent effect on differentially induced apoptosis and necrosis, i.e., a low-dose IR induces apoptosis, while a high-dose IR causes necrosis [57,58].

Similar to the present study, EANT induces a mixture of necrosis and apoptosis at a low concentration, but it generates dominant apoptosis at a high concentration. During the concentration changes, the necrosis and apoptosis switches happen in EANT-treated oral cancer cells.

Since EANT induces oxidative stress, the role of oxidative stress needs to be validated. Following a pretreatment with the oxidative stress inhibitor NAC, the annexin V-detected apoptosis is switched to necrosis at both low and high concentrations of EANT in oral cancer cells (Figure 3). Similarly, NAC suppresses apoptosis-related pancaspase activity at low and high concentrations of EANT (Figure 4), suggesting that NAC suppresses apoptosis. Therefore, EANT differentially regulates necrosis and apoptosis in an oxidative stress-dependent manner.

Necrostatin-1 (NEC1) is the receptor-interacting serine/threonine-protein kinase 1 (RIPK1) inhibitor and is, therefore, commonly used to suppress necroptosis [21]. The annexin V/7AAD-defined necrosis is changed after NEC1 treatment. At low and high concentrations of EANT, the necrosis population (%) in the mixture of necrosis and apoptosis is decreased after NEC1 treatment. These results suggest that this observed necrosis may be the necroptosis type. It warrants a detailed investigation of the detection of necroptosis expression and signaling in the future.

As expected, the apoptosis inhibitor ZVAD slightly decreased the annexin V-detected apoptosis population (Figure 3) and pancaspase activity (Figure 4) in oral cancer cells, following a low concentration of EANT treatment. While at high concentration, EANT induced dominant annexin V-detected apoptosis and could not be decreased by ZVAD. However, the pancaspase activity of CAL 27 cells but not of Ca9-22 cells was suppressed by ZVAD at a high concentration of EANT. Therefore, different cell types show differential regulation of caspase activation.

### 4.4. EANT Triggers DNA Damages in Oral Cancer Cells

In addition to apoptosis, drugs with the oxidative stress-generating ability also show the potential for inducing DNA damage [48]. For example, withaferin A causes oxidative stress and DNA damage (γH2AX and 8-OHdG) [59]. Similarly, EANT shows γH2AX and 8-OHdG inductions in oral cancer cells in a time- and dose-dependent manner (Figure 8 and Figure 9). γH2AX is the phosphorylated form of H2AX. It is noted that these DNA damage detections were based on specific antibodies to these γH2AX and 8-OHdG targets. However, the non-phosphorylated H2AX cannot be measured for observation the total H2AX protein level to detect the interchange between non-phosphorylated H2AX and γH2AX. It warrants further examination of these two DNA damage markers using different methods such as ELISA and Western blotting analysis to provide a comprehensive investigation for EANT-induced DNA damage.

### 4.5. The Role of Oxidative Stress as an Anti-Oral Cancer Mechanism of EANT

The role of oxidative stress for necrosis and apoptosis switches was discussed in Section 4.3. NAC recovers the EANT-induced antiproliferation and inhibits subG1 accumulation, oxidative stress generation (ROS, MitoSOX, and MMP), and DNA damage (γH2AX and 8-OHdG). These results suggest that oxidative stress plays a central role in regulating necrosis and apoptosis switches and DNA damage to antiproliferation of EANT in oral cancer cells.

## 5. Conclusions

The anti-oral cancer effect of ethyl acetate *Nepenthes* extract (EANT) was investigated here for the first time. We report in this study that EANT exhibits oxidative stress-dependent selective killing in several oral cancer cell lines without showing cytotoxicity to normal oral cells. EANT induces several oxidative stress changes and DNA damage, triggers a mixture of necrosis and apoptosis, and partly switches to each other depending on the EANT concentrations. EANT also upregulates antioxidant signaling in response to EANT-induced oxidative stresses in oral cancer cells. Modulating oxidative stress can differentially regulate necrosis and apoptosis switches in oral cancer cells. EANT-induced oxidative stress, DNA damage, and necrosis/apoptosis switches are suppressed by NAC pretreatment. Therefore, EANT selectively kills oral cancer cells by oxidative stress-dependent necrosis/apoptosis switches and DNA damage in oral cancer cells.
